# Biomolecules and Biomarkers Used in Diagnosis of Alcohol Drinking and in Monitoring Therapeutic Interventions

**DOI:** 10.3390/biom5031339

**Published:** 2015-06-29

**Authors:** Radu M. Nanau, Manuela G. Neuman

**Affiliations:** 1 *In Vitro* Drug Safety and Biotechnology, University of Toronto, Toronto, ON M5G 0A3, Canada; E-Mail: radu.nanau@utoronto.ca; 2 Department of Pharmacology and Toxicology, Faculty of Medicine, University of Toronto, Toronto, ON M5G 0A3, Canada

**Keywords:** alcohol, drinking, breath test, ethyl glucuronide, ethyl sulfate, phosphatidylethanol, fatty acid ethyl esters, carbohydrate deficient transferrin, biomarkers

## Abstract

*Background:* The quantitative, measurable detection of drinking is important for the successful treatment of alcohol misuse in transplantation of patients with alcohol disorders, people living with human immunodeficiency virus that need to adhere to medication, and special occupational hazard offenders, many of whom continually deny drinking. Their initial misconduct usually leads to medical problems associated with drinking, impulsive social behavior, and drunk driving. The accurate identification of alcohol consumption via biochemical tests contributes significantly to the monitoring of drinking behavior. *Methods:* A systematic review of the current methods used to measure biomarkers of alcohol consumption was conducted using PubMed and Google Scholar databases (2010–2015). The names of the tests have been identified. The methods and publications that correlate between the social instruments and the biochemical tests were further investigated. There is a clear need for assays standardization to ensure the use of these biochemical tests as routine biomarkers. *Findings:* Alcohol ingestion can be measured using a breath test. Because alcohol is rapidly eliminated from the circulation, the time for detection by this analysis is in the range of hours. Alcohol consumption can alternatively be detected by direct measurement of ethanol concentration in blood or urine. Several markers have been proposed to extend the interval and sensitivities of detection, including ethyl glucuronide and ethyl sulfate in urine, phosphatidylethanol in blood, and ethyl glucuronide and fatty acid ethyl esters in hair, among others. Moreover, there is a need to correlate the indirect biomarker carbohydrate deficient transferrin, which reflects longer lasting consumption of higher amounts of alcohol, with serum γ-glutamyl transpeptidase, another long term indirect biomarker that is routinely used and standardized in laboratory medicine.

## 1. Introduction

Alcoholism and alcohol misuse encompass a spectrum of injury that affects all the organs and tissues of the body [1,2]. It may represent the oldest form of injury known to mankind. Alcoholic beverages existed at least as early as 10,000 BC [3] and liver diseases related to its use have been recognized for almost as long [4]. To this day, alcohol remains a major cause of diseases worldwide [5]. From the medical point of view, alcohol leads to organ injury. Unfortunately, many individuals who misuse alcohol become symptomatic only when severe disease is already present. Patients with alcoholic problems often have coexisting dysfunction such as cardiomyopathy, skeletal muscle wasting, neuropathies, pancreatic dysfunction and parotid gland enlargement [1]. The pharmacokinetics of alcohol determine the time course of ethanol concentration in blood after the ingestion of an alcoholic beverage and the degree of exposure of organs to its effects. The interplay between the kinetics of absorption, distribution and elimination is thus important in determining the pharmacodynamic responses to alcohol. There is a large degree of variability in alcohol absorption, distribution and metabolism, and elimination rate as a result of both genetic and environmental factors. The between-individual variation in alcohol metabolic rates is, in part, due to allelic variants of the genes encoding the alcohol metabolizing enzymes such as alcohol dehydrogenase (ADH) and aldehyde dehydrogenase (ALDH) [6]. The ALDH2*2 and the ADH2*2 alleles, as well as the c2 allele of the cytochrome p450 2E1 (CYP2E1) gene are unique to Orientals. This prompted Sun *et al.* [7] to analyze their involvement in the drinking behavior in 322 middle-aged Japanese men. The ALDH2*2 allele showed a protective effect against a high level of alcohol consumption and problem drinking behavior, as determined by the Kurihama Alcoholism Screening Test. The ADH2*2 allele, present in 95% of individuals, also exhibited a suppressive effect on alcohol consumption. In contrast, the c2 allele of CYP2E1, present in 40% of individuals, was associated with greater alcohol consumption [7].

Possible factors that affect the development of alcohol-related injury include the dose, duration and type of alcohol consumption, drinking patterns, sex, and ethnicity [8,9]. In addition, there are associated risk factors such as obesity, iron overload, concomitant infections, genetic factors, as well as the interaction between therapeutics with alcohol [10,11,12,13,14]. In a study looking at the effect of alcohol consumption on isoniazid therapeutic efficacy in tuberculosis, a direct relationship between the self-reported amount of alcohol consumption and the incidence of hepatic injury was noted. This association becomes clearer when considering that CYP2E1, which metabolizes isoniazid, is induced by ethanol [15]. Environmental and occupational hazards combined with alcohol consumption have to also be taken in consideration. In miners for example, a combination of alcohol abuse and arsenic exposure has been blamed for the occurrence of cirrhosis [16]. In addition, other known hepatotoxins are dangerous in combination with alcohol consumption [17].

Geographic variability exists in the drinking patterns throughout the world [18,19]. Approximately two-thirds of adult Americans consume alcohol [20]. The majority drinks small or moderate amounts, and do so without evidence of clinical disease [21,22,23]. A subset of drinkers consumes excessive amounts of alcohol, develops physical tolerance and withdrawal, and is diagnosed with alcohol dependence. On the other hand, alcohol abusers and problem drinkers engage in harmful alcohol consumption, defined by the development of negative social and health consequences such as unemployment, loss of family, transmission of infectious diseases, organ damage, and accidental injury [24,25,26]. The burden of alcohol-related disease is highest in the developed world, where it may account for as much as 9.2% of all disability-adjusted life years [27,28,29,30]. Even in developing regions of the world, alcohol accounts for a major portion of global disease burden, and is projected to take on increasing importance in those regions over time [26,31]. Alcohol-attributable mortality and burden of disease in Disability-Adjusted Life Years focused on estimating the burden attributable to alcohol consumption, including estimates of exposure (average volume of alcohol consumption and drinking patterns) and determination of risk relations. Moreover, using these estimates to determine alcohol-related burden of disease such as mortality, years of life lost due to premature mortality or due to disability, and disability adjusted life years are important methodologies. The spectrum of laboratory findings in individuals with alcoholic problems can delineate the specific amount and the period when alcohol was consumed. Rehm *et al.* [29] modelled the impact of alcohol dependence on mortality burden and showed that alcohol consumption can affect the available treatment.

### Diagnostic Screening for Alcoholism

The relationship between alcohol consumption and biomarkers was determined in healthy volunteers who consumed controlled levels of alcohol. Reference levels of biomarkers of alcohol consumption are obtained in known teetotalers and individuals required to abstain from alcohol. Biomarkers can be correlated with alcohol consumption patterns in social drinkers. Finally, biomarkers are measured in individuals with alcohol abuse problems, and are used to monitor progress during alcohol withdrawal treatment and potential relapse. The various cut-off values used to delineate different drinking patterns must be considered in light of the specific method used in each study, based on validated methods and manufacturer recommendations for each assay.

Several screening questionnaires exist to establish the link between behavior and diagnosis of alcoholism, including the Kurihama Alcoholism Screening Test [6], the CAGE questionnaire [32,33], the Michigan Alcoholism Screening Test, and the Alcohol Use Disorders Identification Test (AUDIT) [34]. The Michigan Alcoholism Screening Test is a longer test with 25 questions, making it less appealing as a screening tool [35,36]. There have also been comparisons between these methods [37,38]. The AUDIT, developed by the World Health Organization, is a shorter tool with 10 questions aimed to avoid cultural and ethnic bias. The degree to which these questionnaires have been validated varies, and their performance in selected populations also influences their accuracy [39]. In special circumstances such as during evaluation for liver transplantation, in legal cases, or during alcohol withdrawal therapy, there is a need for specific, measurable analysis in patients who deny alcohol intake.

## 2. Materials and Methods

The present systematic review is based on data collected from various recent studies dealing with various biomarkers used to assess alcohol consumption patterns. A PubMed search (2010–2015) was performed using the name of each biomarker (breath test, ethyl glucuronide, ethyl sulfate, phosphatidylethanol, fatty acid ethyl ester and carbohydrate-deficient transferrin) and the term “alcohol”. This was further supplemented by a Google Scholar search.

## 3. Breath Test

The incidence of alcohol-impaired driving was 1.29% in a sample of drivers receiving random breath testing around the city of Barcelona. Trends towards a higher incidence of impaired driving were noted on weekends, during the night, among men, and among drivers traveling with at least one passenger [40]. The incidence of self-reported alcohol consumption (in the preceding 6 h) was 8.3% in a Brazilian sample. This rate was not corroborated by breath teats results due to a low proportion of drivers agreeing to this test [41]. In the context of alcohol consumption, a breath test measures the alcohol level present in exhaled air. The breath alcohol concentration (BrAC) is then used to estimate the blood alcohol concentration (BAC). In cases in which driving offenses are committed, it is beneficial to measure the BAC at the earliest time possible. In most cases, the breath test provides the quickest results. A BAC of >0.05% will result in driver license suspension in the Canadian province of Ontario [42]. An additional blood sample collected at the time of the offence could provide better measurements of BAC. These two techniques can be employed to complement one another [43]. A breath test can further be used in the context of an ignition interlock device that measures the driver’s BAC in individuals with a history of driving under the influence (DUI). The vehicle will not start if the driver’s BAC, as measured by an in-car alcohol breath screening device, exceeds a pre-set limit (e.g., 20 mg alcohol/100 mL blood in the province of Ontario) [42,44]. Prevention programs are also in place. For example, trucks and public vehicles such as buses may be equipped with interlocks that require a breath sample from the operator at the beginning of travel [44]. Moreover, a recent study has shown a poor relationship between how intoxicated many individuals perceive themselves to be and their actual BAC (assessed via breathalyzer), showing the importance of such devices [45].

A mean peak BrAC of 65 ± 19 mg/dL was identified between 20-35 min after alcohol ingestion in a sample of volunteer social drinkers [46]. Among individuals involved in traffic accidents, being BAC positive (≥0.01%, measured in blood or estimated from breath test) was significantly associated with death compared to being BAC negative (*p* < 0.0001) [47]. In order for blood and breath analysis to be interchangeable with each other, the relationship between BAC and BrAC needs to remain stable at all time points. However, the relationship between the two is variable during the absorption stage, and stabilizes during the post-absorptive stage, 60–90 min after alcohol ingestion [48].

A high correlation was found between BrAC and BAC in a sample of healthy volunteers with no history of alcoholism (r = 0.983, 97% sensitivity, 93% specificity) [49]. However, this data is based on means obtained from a large cohort, in which the BAC/BrAC ratios for each individual show a high degree of heterogeneity. When taking into account individual cases, a poor correlation between BrAC and BAC is observed [50]. The breath test thus represents a poor estimate of BAC in real-world situations. Of particular interest are drivers accused of DUI, in which a BAC of ≥0.08%, estimated from the results of the breath test, leads to immediate arrest. Okorocha [50] argues that basing BAC levels on breath test alone can result in innocent drivers getting arrested (overestimated BAC) and guilty drivers being allowed to walk free (underestimated BAC). Furthermore, Ashdown *et al.* [51] calls into question the sensitivity of some commercially available breathalysers. They measured the specificity, sensitivity, positive predictive value (PPV) and negative predictive value (NPV) of three commercially-available breathalyzer devices in a sample of adults who had consumed alcohol, and compared these results with those obtained from a reference police breathalyzer for the purpose of predicting which of them would be over the Unite Kingdom legal driving limit (BrAC 35 μg/100 mL). According to the reference police device, 18.3% of participants were at or over the legal driving limit, while the personal devices show 89.5%, 94.7% and 26.3% sensitivity, respectively, in detecting those individuals at or over the legal limit, showing that these devices vary considerably, and may falsely suggest that some individuals are safe to drive while in reality they may be too inebriated to do so safely [51].

A BAC/BrAC ratio of 2100:1, which is the standard ratio used in law enforcement, was achieved after 30 min in a sample of healthy volunteers, and this remained relatively stable through almost 3 h post-ingestion. This study used a novel breath analyzer that standardizes BrAC to the alveolar-air water vapour concentration [48]. Using a breath analyzer that allows the measurement of alcohol in free exhalation in a small sample of healthy volunteers who drank 0.6 g alcohol/kg body weight, the BAC/BrAC ratio decreased over time (3318 ± 1657 at 2 min, 2514 ± 429 at 5 min, 2311 ± 225 at 10 min, 2246 ± 140 at 15 min, 2089 ± 99 from 30–167 min), with a mean ratio of 2251 ± 46 during the post-absorptive phase. A very good correlation between BrAC ×2251 and arterial BAC was observed (r = 0.998, *p* < 0.001), yet arterial BAC-time profiles of individual patients show a great degree of variability even beyond the 30 min mark when the BAC/BrAC ratio stabilized. In contrast, the correlation between venous BAC and BrAC was poor, with a ratio that fluctuated between 1834 and 3259 [52]. A study conducted in 88 hospitalized patients (35 women and 53 men) shows that estimating BAC from BrAC (2100:1 ratio) would lead to underestimation of venous BAC by 26% compared to the actual measured values [53]. Even using a conversion factor of 2260 led to underestimation of venous BAC by 15% compared to the actual measured values in a sample of drivers [54].

The breath test was a poor estimate of BAC in a sample of bar patrons. Generally, the breath test overestimated BAC in patrons who consumed alcohol only at the bar, and underestimated BAC in patrons who also consumed alcohol before getting to the bar [55]. The presence of mouth alcohol was shown to contaminate BrAC readings [56]. In a different survey, 90.9% of 227 British students attending pub crawls reported drinking prior to arriving at the bar. The median alcohol consumption was 10.0 alcohol units (80 g ethanol) at the time of interview, and it was estimated to exceed 16 by the end of the event. Median BAC among drinkers at the time of interview, measured as BrAC using breathalyser tests and then converted to BAC, was 0.10%. A high BAC was associated with not consuming food in the 4 h prior to interview (OR 1.2, *p* < 0.01), a longer time spent drinking (OR 1.4, *p* < 0.01), and the number of drinks consumed per hour (OR 1.2, *p* < 0.01) [57]. Increasing levels of intoxication, as assessed by breath test, were noted late at night and in the early morning within night-time entertainment districts in an Australian study [58]. Consuming energy drinks was found to have no influence on the subjective (self-reported) level of drunkenness or the objective (assessed using a breath test) intoxication in a sample of Dutch bar patrons. Similarly, consumption of energy drinks did not influence alcohol consumption [59].

An overall strong, positive correlation between the cumulative AUDIT-C score and BrAC reading (r = 0.416, *p* = 0.001) shows a strong correlation between a qualitative measurement of long-term hazardous drinking and current drinking [60]. A breath test did not show sufficiently sensitivity in identifying self-reported heavy drinking in a cross-sectional sample of alcohol-dependent patients [61].

Relapse is routinely measured in patients under alcohol dependence treatment [62]. Relapse was assessed by ethyl glucuronide (EtG) in urine, breath alcohol tests and self-reports in outpatients undergoing long-term alcohol dependence treatment. The percentage of patients showing alcohol relapse was 1.1% by self-report, 4.4% by breath test (range: 0.06–2.60 g alcohol/L), and 37.7% by EtG measurement (mean concentration 47.2 mg/L, range 0.2–1220 mg/L). A good agreement was observed between self-report and breath test, between self-report and EtG, and between breath test and EtG measurement. However, low discrepancies exist, and a high percentage of alcohol relapse cases (93.2%) were only identified by EtG measurement. In contrast, the breath test identified a few cases of alcohol relapse that were not identified by EtG measurement. In these cases, high alcohol concentrations (mean 1.24 g/L) were found, likely suggesting recent alcohol consumption following abstinence [62]. This study shows that each method has its specific timeframe during which it is useful, and multiple methods should ideally be used together in order to identify immediate, short-term or long-term alcohol consumption.

### 3.1. Factors that Affect Breath Test Results

Variability exists in replicate breath alcohol exhalation profiles for one subject collected over a short time interval. There were no age or gender influences, while the breath exhalation volume and breath exhalation time also lacked significant associations [63]. However, the breathing pattern seems to have an effect. These include measuring too early in the expiratory phase, shallow expiration or hyperventilation, or measuring hyperventilation under conditions of chilly ambient temperature. All of these factors give rise to underestimates of BrAC, compared to reference values, while the expired volume is kept constant [64].

The complex exchange of gasses and water in the blood vessels and the mucosa of the airways may further affect the BAC/BrAC ratio [50,65]. Estimating BAC from BrAC is based on the premise that exhaled air reflects the alveolar air alcohol concentration, which is thought to be in direct equilibrium with the blood in the pulmonary circulation. Several factors determine BrAC, most importantly body and lung physiology [66]. During expiration, humidified air at core body temperature loses heat and moisture as it passes through the cooler airway mucosa. Thus, expired air is cooler and drier than alveolar air, as well as containing fewer soluble gases such as alcohol. In addition, alcohol is further lost in the airways, due to the high solubility of the airway tissue to both water and alcohol. Taken together, these suggest that exhaled alcohol levels reflect the concentration of this molecule in the airways rather than in the alveoli [66]. Increases in breath temperature and BrAC were observed with increasing breath volumes, relative to the values for the forced vital capacity. A breath volume of 10% of the forced vital capacity contains around 80% of the end-expiratory breath concentration, and a breath-concentration plateau occurs at around 70% of the forced vital capacity [67]. As BrAC is a function of time and expired air volume, the more of the available lung volume is expired, the higher the BrAC, such that BrAC depicts a more accurate measurement of BAC when an increasing fraction of the available lung volume is expired and BrAC gets closer to the alveolar alcohol concentration.

Body size and lung capacity are important. As some instruments require a volume of exhaled air as high as 1.5 L, an individual with a smaller lung capacity may need to exhale a greater fraction of the available lung volume compared to a person with a higher lung capacity, resulting in a higher BrAC in the exhaled air [66,68]. Breathing patterns also affect BrAC. Hyperventilating or deep breathing lead to lower BrAC, while holding one’s breath leads to higher BrAC than normal breathing. These can be explained by the altered diffusion of alcohol between the expired air and the alveolar mucosa during altered breathing patterns [66,69].

Food has a strong influence on breath alcohol pharmacokinetics. BrAC maximum concentration (C_max_) was highest in fasting subjects (mean 30.5, range 22.5–42 μg/100 mL) and lowest in subjects who consumed a light meal (mean 21.4, range 13.5–32 μg/100 mL) [70]. The time to achieve C_max_ (T_max_) was shortest after a meal (mean 22, range 17–50 min). Alcohol elimination from breath was lower after a meal (mean 5.4, range 3.9–8.5 μg/100 mL/h) than after either fasting (mean 6, range 4.7–7.3 μg/100 mL/h) or a snack (mean 6, range 4.4–8.8 μg/100 mL/h) [70].

An important analytical observation should always be clear. The technical features of the analyzers are different, as are the procedures and the technical ability of the person following them.

### 3.2. Measuring Blood Alcohol Concentration

Xiao *et al.* [71] describe a quantitative method of determining the levels of alcohol in whole blood by headspace gas chromatography-mass spectrometry with good specificity and sensitivity (limit of quantification 39.5 μg/mL and limit of detection 0.4 μg/mL). Headspace gas chromatography/flame ionization detection was also used in a retrospective study to determine BAC in public and private drivers involved in traffic accidents [72]. Headspace gas chromatography/flame ionization detection was also used for post-mortem analysis of blood samples in a large sample of traffic accident victims. This study showed that alcohol was often a contributing factor in traffic accidents (judged to be positive when measured to exceed 0.5 g/kg) [73]. In another study, Sutlovic *et al.* [74] show that while there is generally good agreement between the BAC measured by headspace gas chromatography/flame ionization detection at different time points following different storage periods in post-mortem blood samples, variability of up to 10% was observed in some instances, which is unacceptable in precise forensic evidence. This was judged to result mainly from alcohol oxidation during storage, showing that storage methods have a great influence on the alcohol content in blood [74].

A recent study by Bielefeld *et al.* [75] shows that estimating BAC based on the amount of alcohol consumed (using Widmark’s equation) may lead to erroneous estimates, as significant differences were found between the estimated BAC (Widmark’s equation) and the measured BAC (headspace gas chromatography/flame ionization detection) in a sample of elderly volunteers participating in a drinking experiment aiming to achieve a BAC of 0.6 g/kg (Widmark factors used: 0.7 for males, 0.6 for females). The actual BAC was significantly higher than the estimated BAC in both males and females, and this was found to occur due to a large degree of variation in the calculated individual-specific Widmark factors [75].

Using headspace gas chromatography, Mitchell *et al.* [76] show that C_max_, T_max_ and AUC were different after administration of alcohol (0.5 g/kg body weight), as either beer (5.1% *v*/*v*), white wine (12.5% *v*/*v*), or vodka/tonic (20% *v*/*v*), in a small sample of healthy men. Vodka/tonic (20% *v*/*v*) led to the higher C_max_ and AUC, along with the lowest T_max_. There were no significant differences between beer (5.1% *v*/*v*) and white wine (12.5% *v*/*v*). Spirits resulted in higher exposure than beer or wine, while beer was associated with a lower, more delayed exposure [76].

### 3.3. Ethyl Glucuronide and Ethyl Sulfate in Urine

EtG (ethyl β-D-6-glucuronide) and ethyl sulfate (EtS) represent direct biomarkers of alcohol use. EtG is a minor non-oxidative metabolite of alcohol that forms in the liver through the conjugation of ethanol and glucuronic acid [77]. This reaction is carried out by members of the uridine 5'-diphospho-glucuronosyltransferase family of enzymes, using uridine diphosphate glucuronic acid as a cofactor. Multiple uridine 5'-diphospho-glucuronosyltransferases carry out this reaction, such that they compensate for polymorphisms in one another [78]. However, conversion of alcohol to EtG was recently shown to be influenced by the interaction between uridine 5'-diphospho-glucuronosyltransferases and nutritional components [79].

EtG can be detected in various body fluids, tissue and hair beginning a few hours after alcohol consumption and it remains detectable for up to 80 h after the complete elimination of alcohol from the body [80,81,82]. It can be detected in the blood for up to 36 h and in the urine for up to 5 days, long after alcohol got eliminated [77,83]. EtS is another minor, direct alcohol metabolite, produced through conjugation with sulfate. This reaction is carried out by cytosolic sulfotransferase enzymes [84]. EtG is primarily used to detect heavy alcohol use. Although only a relatively low amount of alcohol is eliminated by glucuronidation, EtG is an important biomarker that can determine alcohol consumption. The presence of EtG indicates recent alcohol use, even if there is no detectable alcohol in the body [83]. Urine EtG and EtS have been identified as important markers of recidivism in a large sample of drivers charged with DUI in a Canadian study [85].

A recent alcohol challenge study (doses calibrated to achieve blood concentrations of 20, 80 or 120 mg/dL) shows that urine EtG was always detectable at the 100 and 200 ng/mL cut-offs 12 h after ingestion. At 24 h, the sensitivity associated with these cut-offs was low following ingestion of the low does. The sensitivity of the assay was low at 24 h regardless of dose. There was generally good correlation between urine EtG and EtS. This report showed that light drinking can be detected through urine EtG analysis during the first 24 h [86]. Results of recent studies correlating urine EtG and EtS with alcohol consumption patterns are shown in Table 1 [87,88,89,90,91,92,93].

Positive EtG (>0.1 mg/L) and EtS (>0.05 mg/L) can be measured in healthy volunteers drinking 1–2 drinks for up to 24 h. Among patients under withdrawal treatment, the highest urine EtG and EtS levels were obtained at the first sample, and decreased with time and repeated sampling [94]. The kinetics of EtG and EtS formation and elimination were assessed in a small sample of healthy volunteers after consuming 4 or 8 units of alcohol (40 or 80 mL) [95]. Median EtG C_max_ was 0.4 ± 0.3 μg/mL in serum and 3.5 mg/h ± 1.2 mg/h in urine after 4 units, achieved after 2.0 ± 0.8 h in serum and 3.0 ± 1.0 h in urine (T_max_). The corresponding C_max_ values for EtS were 0.2 ± 0.1 μg/mL in serum and 1.3 ± 0.6 mg/h in urine, with T_max_ 1.0 ± 1.0 h and 2.0 ± 0.5 h, respectively [95]. After 8 units, EtG C_max_ was 1.3 ± 0.4 μg/mL in serum and 10 ± 3.4 mg/h in urine, with T_max_ 4.0 ± 1.8 h and 4.0 ± 2.0 h, respectively. EtS C_max_ was 0.6 ± 0.1 μg/mL in serum and 3.5 ± 1.1 mg/h in urine, with T_max_ 3.0 ± 1.0 h and 3.0 ± 1.0 h, respectively. The EtG/EtS ratio increased as a function of time after alcohol administration in both serum and urine samples for up to 6 h. This occurred to a lesser extent after 8 units of alcohol than after 4 units [95]. A dose-effect relationship between alcohol ingestion and EtG C_max_ was observed in another sample of healthy volunteers. EtG levels in urine were higher than in blood or saliva, suggesting that EtG measurements are most sensitive in urine [96].

**Table 1:** Ethyl Glucuronide and Ethyl Sulfate in Urine.

Study	Biomarker and Method	Study Population and EtG and EtS Levels	EtG, EtS/Diagnostic Performance
87	EtG and EtS Ultraperfor-mance LC-electrospray tandem MS	Healthy volunteers with documented alcohol consumption during past 5 days; EtG between 0.5–101.9 µg/mL (mean 10.9, median 1.4) and EtS between 0.1–37.9 µg/mL (mean 3.6, median 0.3) in subjects who consumed alcohol the day before sampling	n/a
88	EtG and EtS Method not specified	Active duty service members receiving addiction treatment; Paired results negative (<100mg/mL and <50 mg/mL, respectively) in 78.9%; Paired results positive in 10.2%. Only one of EtG (2.3%) or EtS (8.6%) positive in remaining 10.9% of samples, with the other one negative	n/a
89	EtG Method not specified	Active duty service members receiving addiction treatment 17.2% of samples tested positive (>250 ng/mL); Positive specimens ranged between 260–330000 ng/mL: 42.2% ranged between 1000–9999 ng/mL; 28.9% exceeded 10000 ng/mL; Among subjects who tested positive at baseline, 64.4% also tested positive at a future time point	22.8% PPV and 85.5% NPV for AUDIT score with respect to initial EtG test
90	EtG-Immuno-assay LC-MS/MS	Orthotropic liver transplant recipients; Positive in 71.4% of patients	89.3% sensitivity, 98.9% specificity, 89.3% PPV, 98.9%; NPV (OR 761.1, *p* < 0.001) detect relapse at a cut-off of 0.5 mg/L
91	EtG and EtS LC-MS/MS	Hepatology clinic patients Median EtG 1918 ng/mL (IQR 556->10000) and median EtS 459 ng/mL (IQR 90–2981) among subjects testing positive	76% sensitivity, 93% specificity, 81% PPV; 91% NPV for positive urine EtG (>100 ng/mL) *vs.* past 3 days drinking; 82% sensitivity, 86% specificity, 70% PPV, 93% NPV for positive urine EtS (>25 ng/mL) *vs.* past 3 days; 70% sensitivity, 99% specificity, 97% PPV, 85% NPV for positive urine EtG *vs.* past 7 days drinking; 73% sensitivity, 89% specificity, 80% PPV, 85% NPV for positive urine EtS *vs.* past 7 days drinking
92	EtG and EtS UPLC-MS/MS	Female sexual assault victims EtG 0.34–1123 mg/L and EtS 0.18–322 mg/L in positive samples	94% sensitivity, 100% specificity, 100% PPV; 79% NPV for positive EtG and EtS in urine *vs.* self-reported alcohol intake; 42% sensitivity, 100% specificity, 100% PPV, 28% NPV for positive blood alcohol (37–280 mg/dL) to self-reported alcohol intake
93	EtG and EtS LC-MS/MS	Alcohol-dependent patients in withdrawal EtG ranged from 90–850 pg/mL (normalized to 100 mg/dL creatinine); EtS ranged from 20–280 pg/mL	n/a

EtG—ethyl glucuronide; EtS—ethyl sulfate; LC—liquid chromatography; LC-MS/MS—liquid chromatography-tandem mass spectrometry; MS—mass spectrometry; NPV—negative predictive value; OR—odds ratio; PPV—positive predictive value.

Urinary EtG correlates well with EtS [87,92,93]. Both EtG and EtS were below the cut-off value among subjects who denied alcohol consumption, and were generally undetectable (<0.1 µg/mL) in subjects who reported only one drink on the day before sampling. EtG (r = 0.448, *p* < 0.02) and EtS (r = 0.406, *p* < 0.04) show modest correlation with the number of drinks [87]. In a different study, the minimum EtG and EtS were both found in the same individual, as were the maximum EtG and EtS. Both EtG and EtS levels decreased rapidly in alcohol-dependent patients during withdrawal. EtG and EtS were detectable on day 8 in one patient only, both of which were present in the same individual [93].

EtG and EtS in urine can help either prove or disprove self-reported alcohol consumption patterns in various populations in which abstinence is encouraged. Self-reported past 3 days, alcohol consumption was significantly related to the EtG and EtS concentrations in urine in patients from hepatology clinics (r = 0.94, *p* < 0.001) [91]. Urine EtG was identified as the strongest marker of alcohol consumption in a sample of liver transplant recipients and liver transplant candidates. Urine EtG levels were strongly correlated with the amount of alcohol consumed (*p* < 0.001), and as such it can be used to detect alcohol consumption (89.2% sensitivity, 98.8% specificity, 97.1% PPV and 95.4% NPV at cut-off >500 ng/mL). This biomarker was useful in predicting alcohol consumption in subjects with either positive or negative AUDIT results [97].

Urine EtG tested positive in 29.3% of patients undergoing treatment for alcohol use disorder, of which 45.5% admit recent alcohol consumption and 22.7% have positive breath tests [98]. While superior to a breath test in identifying recent alcohol consumption, urine EtG still generally detects only moderate to high alcohol consumptions in the past 2 days prior to sample collected [99].

#### 3.3.1. Discussion

Urinary EtG and EtS were used to estimate relapse in outpatients treated for alcohol-related problems. Alcohol consumption is higher during the weekend than throughout the week in outpatients [62,100]. A good correlations exists between the quantity of self-reported drinking in the 3 days prior to each sample collection and urinary EtG (r = 0.662, *p* < 0.001) and EtS (r = 0.716, *p* < 0.001) levels. No recent drinking was self-reported in patients with samples negative for EtG or EtS [101]. Urinary EtG and EtS can identify long-term alcohol use in the presence of other markers such as phosphatidyl-ethanol (PEth) and/or carbohydrate-deficient transferrin (CDT), or can indicate occasional alcohol use when present on their own [102]. Serial testing led to a significant decline in positive samples over time (*p* = 0.017) among active duty service members receiving addiction treatment. EtG positivity generally correlates poorly with the AUDIT score [89].

Urinary EtG is an important biomarker for assessing alcohol abstinence in orthotropic liver transplantation [90]. Only 3.6% of potential recipients admitted alcohol consumption in a sample of 141 patients, despite 19.8% being positive for at least one alcohol biomarker (urinary EtG, ethanol, methanol, CDT, aspartate aminotransferase (AST), alanine aminotransferase (ALT), γ-glutamyl transpeptidase (γ-GTP) and mean corpuscular volume (MCV)) at any visit. Of these, urinary EtG was the best predictor of alcohol consumption, and increased detection of alcohol consumption compared to other biomarkers (*p* < 0.001) [90]. The prevalence of urinary EtG and EtS was higher in patients with alcoholic liver disease than in patients with other liver conditions (20% *vs.* 5%, *p* = 0.04) [103]. The presence and levels of urine EtG and EtS are also related to the incidence of failing an ignition interlock device BAC test among drivers convicted of DUI [104].

Furthermore, during post-mortem analysis, 68% of individuals with a history of alcohol abuse were found to have a positive BAC (median 1.15‰, range 0‰–3.3‰). EtG concentrations in urine were significantly higher in individuals with a history of alcohol abuse during post-mortem analysis than in individuals without a documented history of alcohol abuse (339 ± 389 mg/L, *p* < 0.001) [105].

#### 3.3.2. Factors Affecting Urine EtG

As urine EtG and EtS are short-term biomarkers, false negatives may arise due to low alcohol intake (<3 drinks) or a long period between alcohol intake and sample collection (>16 h) despite self-reported alcohol intake [92]. On the other hand, urine EtG and EtS tests are so sensitive to the presence of alcohol that that they are unable to distinguish between alcohol abstinence and low levels of alcohol consumption, and false positive results may be obtained even from accidental exposure [82,92]. Among subjects with no past 7 days drinking history, positive urine EtG and EtS results likely reflect non-beverage alcohol exposure [91]. Thus, factors unrelated to drinking may further distort results, such that alcohol consumption may be either underestimated or overestimated.

Despite EtG values being slightly lower when measured by enzyme immunoassay, a good correlation was found between enzyme immunoassay and liquid chromatography-tandem mass spectrometry (LC-MS/MS) (r^2^ 0.996 in clinical samples and 0.956 in post-mortem samples), with a strong correlation between EtG and EtS (r^2^ 0.9025, *p* < 0.001, mean EtG/EtS ratio 3.8, median EtG/EtS ratio 3.5) [106]. A good correlation between urine EtG levels measured by a commercially available immunoassay test and a lab-based mass spectrometry test was further observed in a sample of adults with alcohol dependence [107]. A good correlation (r = 0.96–0.98) was further observed between different liquid chromatography-mass spectrometry (LC-MS) methods of measuring EtG in urine [108].

Using dose-adjusted detection times, decreased renal function led to significantly longer detection times for urinary EtG and EtS compared to healthy subjects (*p* < 0.01). C_max_ values were lower, and the detection time of EtG and EtS was correlated to the degree of renal dysfunction. The implication of this is that individuals with decreased renal function may be wrongly suspected of higher or more recent alcohol consumption [109].

Intensive use of mouthwash (4 times/day for 3¼ days) led to EtG and EtS below 500 ng/mL, thus allowing intentional alcohol use to be distinguished from accidental exposure [110,111]. On the other hand, intensive use of an alcohol-based hand sanitizer (120 times/day for 3 days) led to mean EtG levels of 278 ng/mL, with maximum EtG 2001 ng/mL in one subject. The urine concentration of EtG was highest at the end of each study day. EtS levels were lower than EtG levels (<100 ng/mL in all samples). As such, the presence of EtS may allow intentional alcohol use to be distinguished from dermal exposure [112]. A separate study found that transdermal exposure to hand sanitizer does not affect urine EtG levels. However, inhalation of hand sanitizer may increase urinary EtG levels [113].

Non-alcoholic beers (<0.5% alcohol), sauerkraut and matured bananas lead to urine EtG levels >0.1 mg/L for up to 13, 5 and 3.5 h later, respectively [114]. EtG (0.30–0.87 mg/L) and EtS (0.04–0.07 mg/L) were positive after consuming 2.5 L of non-alcoholic beer. EtG were above the abstinence cut-off of 0.1 mg/L. In one subject, overnight accumulation in urine led to high levels of EtG (14.1 mg/L) and EtS (16.1 mg/L) [115]. EtS was positive in urine in subjects consuming non-alcoholic wine (C_max_ 2.15 mg/L). No such relationship was observed for EtG [110]. The consumption of baker’s yeast and sugar led to EtG and EtS levels above the 0.1 mg/L cut-off for abstinence (0.67 and 1.41 mg/L, respectively). Alcohol was not detected in urine [116].

Ethanol glucuronidation is increased by cannabinol in a dose-dependent manner, and is decreased by cannabidiol in a noncompetitive manner. Other common drugs of abuse like morphine, codeine, lorazepam, oxazepam, nicotine or cotinine had no significant effects on ethanol glucuronidation [117]. Age, gender, ethnicity and liver disease severity did not significantly affect the association between past 3 days drinking and urine EtG or EtS [91].

As there are several factors that can lead to low positive urinary EtG tests in individuals who deny drinking, additional tests can differentiate between accidental alcohol exposure and the patient hiding alcohol consumption. In a recent analysis, 55.6% of individuals testing positive for low levels of urinary EtG or EtS denied drinking. Among these, negative PEth test results supported the subjects’ claim of alcohol abstinence and likely suggests accidental exposure in 70.0%, while positive PEth test results contradicted the subjects’ claim in 20.0% [118].

## 4. Phosphatidylethanol

PEth is a phospholipid with two long chain carboxylic acid residues formed only in the presence of ethanol under the action of phospholipase D. A total of 48 PEth homologues were identified in blood samples from an autopsy case, containing 14 to 22 carbon atoms and 0 to 6 double bonds per molecule. The most abundant of these were PEth 16:0/18:1 and PEth 16:0/18:2. PEth 16:0/18:1 is the most common species, and it was about 10 times more abundant than PEth 16:0/16:0 and PEth 18:1/18:1, the other two PEth species routinely analyzed [119]. In all, 17 PEth species were identified in heavy drinkers (>0.001 µM) in a different study, the predominant ones of which were 16:0/18:0, 16:0/18:2 and 18:0/18:1. Only 2 PEth were identified in social drinkers, namely 16:0/18:0 and 16:0/18:2 [120].

Phospholipase D normally catalyzes the hydrolysis of phospholipids to form phosphatidic acid. However, phospholipase D has a higher binding affinity for ethanol than water, resulting in the preferential production of PEth over phosphatidic acid in the presence of even low quantities of alcohol. PEth comprises a group of phospholipids with a common non-polar phosphoethanol head group and two fatty acid moieties [77,82]. PEth has a half-life of approximately 4 days in blood in alcoholic subjects admitted for detoxification, with no correlation to baseline PEth levels [121]. Results of recent studies correlating blood PEth with alcohol consumption patterns are shown in Table 2 [102,122,123,124,125,126,127].

In a small sample of healthy volunteers drinking the equivalent of 1 g/kg alcohol for 5 consecutive days after 3 weeks of abstinence, followed by a further 16 days of abstinence after the drinking episode, the maximum BAC was 0.99–1.83 g/kg (mean 1.32 g/kg), reached after 1–3 h (mean 1.9 h) after the start of drinking. The maximum PEth 16:0/18:1 levels, measured by LC-MS/MS were 45–138 ng/mL 1 h after the start of drinking. PEth was detectable in 90.9% of the sample. Blood PEth levels continued to rise over the following days, peaking at 74–237 ng/mL between days 3 and 6 [128]. Trace levels of PEth 18:1/18:1, 16:0/16:0 and 18:1/16:0 (or 16:0/18:1) were detected in a blood sample collected 3 h post-drinking in another social drinker after a single 60 g alcohol dose, with no PEth detected prior to alcohol consumption after 3 weeks of abstinence [129].

**Table 2:** Phosphatidylethanol in Blood.

Study	Method	Study Population—PEth Levels	Peth—Diagnostic Performance
122	LC-MS/MS	Women of reproductive age, Median PEth 45 ng/mL (range 0–565 ng/mL), PEth undetectable in 71.2% of subjects; PEth detectable in 53.3% of subjects reporting 2 drinks/day	n/a
123	LC-MS/MS (HPLC)	Pregnant women who self-reported alcohol ingestion between 2.5–20 drinks/week; Good correlation between self-reported drinking and PEth; PEth-16:0/16:0, 16:0/18:1 and 18:1/18:1 below the lower limit of quantification (1.5 nmol/L for PEth-16:0/16:0, 3.1 nmol/L for PEth-16:0/18:1 and 1.2 nmol/L for PEth-18:1/18:1) abstinents;	n/a
		PEth-16:0/18:1 positive in all subjects with self-reported alcohol ingestion; PEth-16:0/16:0 (84.6% positive) and PEth-18:1/18:1 (positive 54.8%) of subjects with self-reported alcohol ingestion;	
		Total PEth levels varied between 4.8–182.9 nmol/L among self-reported alcohol ingestion	
124	LC-MS/MS (HPLC)	Pregnant women 34.8% abstainers, 42.3% light drinkers, 4.3% moderate drinkers and 18.7% heavy drinkers before conception; PEth levels correlated with drinks per occasion (*p* < 0.001) and days drinking per week (*p* < 0.001), but not with the time from last ingestion or duration of drinking in the first trimester of pregnancy	PEth concentration increased by 9.5 nmol/L per drink ingested on each occasion, and 5.8 by nmol/L per drinking day/week
125	LC-MS/MS	HIV-positive patients, 66.2% report alcohol consumption, 20.8% report frequent alcohol consumption (≥3 times/week or BrAC >0.1%) 51.9% heavy alcohol consumption (self-reported >42 g for women or >56 g for men), 14.3% report frequent heavy alcohol consumption	87.8%–100% sensitivity and 43.9%–88.5% specificity to determine any alcohol, frequent alcohol, any heavy alcohol or frequent heavy alcohol consumption in 7, 14 or 21 preceding days; Overall 88.0% sensitivity and 88.5% specificity for any alcohol consumption during any of the preceding 21 days
126	LC-MS/MS	HIV-positive patients, 37.3% of 150 blood samples were PEth-positive (≥8 ng/mL);	n/a
		PEth highly correlated with total number of drinking days in last 30 days (*p* < 0.001)	
127	LC-MS/MS	Patients with chronic liver disease 4% of self-reported abstainers, 65% of subjects with <4 drinks/day and 97% of subjects with ≥4 drinks/day were PEth positive (≥20 ng/mL)	79% sensitivity, 90% specificity at cut-off ≥8 ng/mL (any drinking); 73% sensitivity and 96% specificity at cut-off ≥20 ng/mL (any drinking);
			97% sensitivity, 66% specificity for ≥4 drinks/ day at cut-off ≥20 ng/mL; 91% sensitivity and 77% specificity for ≥4 drinks/ day at cut-off ≥80 ng/mL
102	LC-MS	Outpatients treated for alcohol-related problems; Range 0–16.5 µmol/L (mean 2.6), with 70% above the quantification limit (0.1 µmol/L) and 55% above the reference cut-off for alcohol abuse (0.7 µmol/L) at initial assessment; PEth-16:0/18:1 levels decreased from 0–4.7 µmol/L (mean 0.98 µmol/L, median 0.67 µmol/L) at the start of the study to 0–2.3 µmol/L (mean 0.22 µmol/L, median 0.00 µmol/L, *p* < 0.0001) at the end of the study	n/a

BrAC—breath alcohol concentration; LC-MS/MS—liquid chromatography-tandem mass spectrometry; PEth—phosphatidylethanol.

Negative PEth levels are found in teetotalers, while positive PEth levels are found in samples belonging to known alcoholic patients [130]. PEth levels generally decreased in subsequent samples in outpatients treated for alcohol-related problems, with a half-life of 3.5–9.0 days (mean 6.1 days, median 7.0 days) [102,131]. Blood PEth remains detectable for up to 14 days after the last drink in alcoholics admitted for alcohol detoxification [132]. Furthermore, PEth 0.9 µM was detected in a subject with a long-term history of alcohol abuse 9 days after the last drink, showing that PEth can be detected after a relatively long time since stopping drinking in subjects with a history of high alcohol consumption [133].

Blood PEth levels were assessed in two cohorts of HIV-positive patients who were expected to remain abstinent while waiting to start antiretroviral treatment. A high rate of alcohol consumption was found in one these populations, both by self-reporting as well as through positive PEth results [125]. In the other study, 37.3% of samples were PEth-positive (>8 ng/mL), despite over half of these individuals denying alcohol consumption. Men and subjects from lower economic classes were found to be more likely to under-report alcohol consumption [126]. PEth results were strongly correlated with AUDIT-C scores and measurements of alcohol consumption, including binge drinking in a sample of injecting drug users. Interestingly, almost 95% of individuals who did not report alcohol consumption actually tested negative for PEth [134].

PEth levels in blood distinguished between heavy drinkers (>60 g/day) and social drinkers in a meta-analysis (mean 3.897 *vs.* 0.288 μmol/L). As such, PEth is a tool used primarily to identify chronic excessive drinking [135]. Currently, there is no uniformly accepted cut-off level to differentiate between social drinkers (<40 g/day for males and <20 g/day for females), at-risk drinkers (40–60 g/day) and chronic heavy drinkers (>60 g/day), although a threshold of 0.7 μmol/L is sometimes used to classify alcohol-related problems, with blood PEth < 0.7 μmol/L generally consistent with low or moderate alcohol consumption in the two weeks prior to sample collection [131,136].

As the formation of blood PEth is specifically dependent on blood alcohol levels, a strong correlation exists between alcohol consumption and blood PEth levels [131,133]. PEth tests can monitor alcohol consumption, can help identify early signs of harmful alcohol consumption, and can help track cases of alcohol abuse or dependence [131]. PEth has 99% sensitivity for detecting excessive alcohol consumption with a cut-off of >0.22 µM [133].

### 4.1. Discussion

PEth was associated with ignition interlock devices BAC test failure. Higher PEth levels were found in individuals with a higher risk of interlock BAC failure (1.45 ± 1.17 µmol/L) compared to the low risk group (0.61 ± 0.61 µmol/L) [104]. LC-MS/MS (limit of detection 0.005 μmol/L) identified positive alcohol consumption in a higher proportion of driver blood samples with failed interlock blood alcohol than high-performance liquid chromatography (HPLC) (limit of detection 0.25 μmol/L). A good correlation was found between the methods. Both methods further identified alcohol consumption in DUI offenders without failed interlock tests. Overall, 88.5% of samples were positive by LC-MS/MS and 71.2% were positive by HPLC [137]. Interestingly, while zero failed interlock BAC tests suggests an absence of drinking and driving behavior, negative PEth suggests absence of drinking. Therefore, interlock BAC tests and PEth tests distinguish between drinking, and drinking and driving behavior [137].

PEth analysis is a measure of sobriety in alcohol-dependent subjects entering detoxification. Using a limit of quantification of 0.22 µmol/L, blood PEth correlates well with alcohol consumption during the 7 days prior to entering alcohol detoxification (range 0.63–26.95 µmol/L at day 1, mean 6.22 µmol/L, median 4.70 µmol/L). The sensitivity of PEth decreases with passing time since admission from 100% at day 1, to 92.5, 76 and 64.3% at days 7, 14 and 28, respectively. Gender does not influence PEth levels [138].

### 4.2. Factors Affecting Blood Phosphatidylethanol

A trend towards higher PEth levels with increasing alcohol consumption levels is reported in a recent review of published data. Blood PEth is generally undetectable in abstinent subjects and low in the general population. PEth is strongly affected by the subject’s drinking pattern. The odds of detecting PEth in blood were associated with the average daily alcohol consumption pattern, especially the cumulative amount of alcohol consumed in a period of time (1–2 weeks) [139]. Blood PEth levels do not correlate with the number of heavy drinking days, the number of days during which any alcohol was consumed, or days since the last drink. PEth levels were not correlated with the average number of drinks/week (r < 0.05, *p* > 0.05). A trend towards an association with days since the last heavy drinking day was however observed, with PEth levels significantly correlated with heavy drinking during the preceding 1–4 days (*p* < 0.001) but not during the preceding >5 days (*p* > 0.2) [122,123]. PEth levels were particularly high in DUI subjects (median 0.5, mean 0.7 µmol/L), especially high-risk DUI subjects (median 1.0, mean 1.5 µmol/L), as well as in alcohol clinic outpatients (median 2.9, mean 3.4 µmol/L in lower risk outpatients and median 7.5, mean 7.5 µmol/L in high-risk inpatients) [44]. PEth was not detected in a sample of pregnant women with low, infrequent alcohol consumption [140].

A strong correlation was found between blood PEth and urine EtG and EtS, all of which represent markers of recent alcohol consumption. In contrast, blood PEth was not associated with CDT and γ-GTP levels [104]. PEth is not related to other biomarkers in subjects undergoing alcohol withdrawal [93]. Liver diseases or hypertension do not influence blood PEth levels [139].

## 5. Ethyl Glucuronide and Fatty Acid Ethyl Esters in Head Hair

Hair EtG is an important marker of long-term alcohol consumption [141]. A linear correlation between hair EtG levels and the amounts of alcohol consumed in alcohol-dependent individuals was recognized in a recent study [142]. The Society of Hair Testing identifies EtG (cut-off 30 pg/mg in the 0–3 cm proximal segment) and fatty acid ethyl esters (FAEE) (cut-off 0.5 ng/mg in the 0–3 cm proximal segment or 1.0 ng/mg in the 0–6 cm proximal segment) as direct alcohol consumption markers that can be used to determine excessive alcohol consumption. These cut-offs are considered to correspond to chronic excessive alcohol consumption of >60 g/day for several months. The concomitant use of these two molecules is recommended in order to prevent false positive or false negative results with either biomarker. A 3 cm segment of hair corresponds roughly to a 3-month history of drinking pattern [143,144]. Results of recent studies correlating hair EtG with alcohol consumption patterns are shown in Table 3 [130,145,146,147,148,149,150,151,152,153,154,155,156,157,158].

### 5.1. Ethyl Glucuronide

Hair EtG analysis showed excessive alcohol consumption for approximately 17 months prior to sampling in a murder victim (>170 pg/mL), in a driver whose license was reinstated as result of a lack of regular alcohol consumption (<10 pg/mL), and in another driver whose license was suspended as result of regular alcohol consumption (>30 pg/mL). Hair EtG levels were undetectable or <1.0 pg/mL in 10 teetotalers [159]. In a meta-analysis, the hair EtG concentrations in social drinkers (mean 7.5 pg/mg, 95% CI 4.7–10.2, *p* < 0.001), heavy drinkers (mean 142.7 pg/mg, 95% CI 99.9–185.5, *p* < 0.001) and deceased subjects with a known history of chronic excessive drinking (mean 586.1 pg/mg, 95% CI 177.2–995.0, *p* < 0.01) were higher than the values in teetotalers (<7 pg/mg, with slight overlap with social drinkers) [160]. Alcohol consumption of 16 g/day for 3 months did not lead to hair EtG levels higher than the threshold of 7 pg/mg for alcohol abstinence, while no subject consuming 32 g/day had hair EtG in excess of 30 pg/mg consistent with alcohol abuse [161].

### 5.2. Fatty Acid Ethyl Esters

A recent large study conducted in healthy volunteers assessed the relationship between self-reported daily alcohol intake and FAEEs concentration (ethyl myristate, ethyl palmitate, ethyl oleate, and ethyl stearate) in hair with the scope of differentiating alcohol abstinence from moderate (<60 g/day) or excessive drinking (≥60 g/day). Based on the correlations between self-reported daily alcohol intake and FAEEs concentration, this study found that a FAEEs cut-off of 0.5 ng/mg in 3 cm of proximal hair offers the best means of discriminating between social drinking and excessive alcohol consumption [162]. Mean FAEEs levels were 0.87 ng/mg ± 214% in another sample of volunteers, with 0.42 ng/mg ± 114 in non-drinkers or social drinkers, and 1.41 ng/mg ± 186 in alcoholics. FAEEs in hair samples show 59.3% sensitivity and 91.0% specificity for heavy drinking at a cut-off level of 0.675 ng/mg [157].

**Table 3:** Ethyl Glucuronide in Head Hair.

Study	Method	Study Population and EtG Levels	Head Hair EtG Diagnostic Performance
145	LC-MS/MS	Volunteers: Positive in 16.1% of samples (30–1200 pg/mg, median 63.5 pg/mg) using a cut-off of 30 pg/mg	n/a
146	GC-MS/MS	Volunteers with a wide range of alcohol consumption patterns	26% sensitivity, 95% specificity, 95% PPV. 23% NPV for drinking of any kind with a threshold of 30 pg/mg; 53% sensitivity, 91% specificity, 73% PPV, 82% NPV for intermediate or high-risk drinking—threshold 30 pg/mg; 58% sensitivity, 86% specificity, 50% PPV, 90% NPV for high-risk drinking - threshold of 30 pg/mg
147	GC-MS/MS	Volunteers assessed according to the Daily Alcohol Self-Monitoring log, EtG > 9 pg/mg associated with alcohol consumption of >20/30 g (at-risk drinkers), EtG > 25 pg/mg associated with alcohol consumption of >60 g (heavy drinkers)	93% sensitivity, 94% specificity, 89% PPV and 96% NPV for teetotalers with a cut-off of 0 pg/mg; 82% sensitivity, 93% specificity, 84% PPV and 92% NPV for at-risk drinkers with a cut-off of >9 pg/mg; 95% sensitivity, 97% specificity, 88% PPV and 99% NPV for heavy drinkers with a cut-off of >25 pg/mg
148	HILIC-MS/MS	Volunteers: 66.7%—regular moderate drinkers based on hair EtG 1.34–82.73 pg/mg	n/a
149	GC-MS	Orthotropic liver transplant candidates	97% specificity, 85% sensitivity, 85% PPV and 89% NPV, with a cut-off of 30 pg/mL (60 g/day) for heavy drinking (>60 g/day)
150	LC-MS/MS	Hepatology clinic patients: Average alcohol consumption higher in subjects with positive hair EtG (median 56 g/day, range 0.3–310 g/day) than among subjects with negative hair EtG (median 3.1 g/day, range 0.1–68.5 g/day) (*p* < 0.001)	92% sensitivity and 87% specificity for detecting self-reported alcohol consumption averaging ≥28 g/day with a cut-off of ≥8 pg/mg
151	LC-MS/MS	Liver transplant recipients (29.8% had underlying alcoholic liver disease); Abstinence or rare drinking in 85.6% (EtG <7 pg/mg); Regular alcohol consumption (>10 g ethanol/day) in 14.4% (EtG 7–30 pg/mg); Excessive chronic consumption (>60 g ethanol/day) in 8.6% (EtG >30 pg/mg)	n/a
152	LC-ESI-MS/MS	Employees with suspected alcohol abuse, Negative in 61.5% (<7 pg/mg); Moderate drinking identified in 20.5% (7–30 pg/mg); Chronic excessive drinking suspected in 17.9% (≥30 pg/mg)	n/a
153	Method not specified	Drivers with zero tolerance: 50.4% shown to be abstainers by undetectable EtG (30pg/mg)	n/a
154	Various hair matrices GC-MS/MS	Subjects assessed for fitness to drive: Scalp hair: mean 79 pg/mg, median 23 pg/mg, (undetectable-1600 pg/mg); Chest hair: mean 63 pg/mg, median 24 pg/mg (range: undetectable-520 pg/mg); Arm hair: mean 87 pg/mg, median 43 pg/mg, (undetectable-880 pg/mg); Leg hair: mean 84 pg/mg, median 30 pg/mg, (undetectable-970 pg/mg); Axillary hair: mean 6 pg/mg, median 4 pg/mg, range from undetectable-20 pg/mg	n/a
155	LC-MS/MS	Subjects claiming abstinence 85.0% of subjects were abstainers (EtG < 7 pg/mg); 10.0% of subjects were social drinkers (EtG 7–30 pg/mg) 5.0% of subjects were chronic excessive drinkers (EtG >30 pg/mg)	n/a
156	LC-MS/MS	Chronic excessive alcohol consumption assessed for driving/firearm license renewal, adoption or liver transplant; EtG < 30 pg/mg in all subjects deemed eligible by a physician; EtG < 7 pg/mL in 7.8%, 7–30 pg/mg in 23.1% and >30 pg/mg in 69.2% of subjects deemed not eligible by a physician	100% specificity and 69% sensitivity at a cut-off of 30 pg/mg for predicting chronic excessive alcohol consumption
157	LC-MS/MS	Mean EtG 77 pg/mg ± 245% in the overall samples, 3.9 pg/mg ± 259% in non-drinkers or social drinkers (60 g/day)	83.3% sensitivity, 97.4% specificity at a cut-off level of 28 pg/mg for an alcohol consumption cut-off of 60 mg/day
158	LC-MS/MS	Patients participating in alcohol treatment or in clinical trials; 68 ± 133 pg/mg in in the overall population; 3.5 ± 1.2 pg/mg in non-drinkers 8.0 ± 9.2 pg/mg in social drinkers; 191 ± 173 pg/mg in heavy drinkers	91.5% sensitivity and 97.4% specificity for chronic heavy drinking, with a cut-off of 30 pg/mg
130	LC-ESI-MS/MS	Post-mortem analysis in subjects with potential alcohol problems Range 0–653 pg/mg; 76% below the cut-off of 30 pg/mg; 9% between 30–50 pg/mg; 15% above 50 pg/mg-considered alcoholics	n/a

EtG—ethyl glucuronide; GC-MS/MS—gas chromatography-tandem mass spectrometry; HILIC-MS/MS Hydrophilic interaction liquid chromatography-tandem mass spectrometry; LC-MS/MS—liquid chromatography-tandem mass spectrometry; NPV—negative predictive value; PPV—positive predictive value.

FAEEs were assessed in a large sample of 1057 autopsy cases (168 social drinkers, 502 alcohol abusers and 387 unknown). Median FAEEs levels were 0.302 ng/mg (range 0.008–14.3 ng/mg) among social drinkers and 1.346 ng/mg (range 0.010–83.7 ng/mg) among alcohol abusers. Based on these findings, the optimal cut-off value for differentiating social drinkers from alcohol abusers was calculated at 1.08 ng/mg [157].

Using cumulative concentrations of ethyl myristate, ethyl palmitate, ethyl oleate and ethyl stearate, FAEEs ranged between 0.11–31 ng/mg (mean 1.77 ng/mg, median 0.82 ng/mg), with 46.3% of samples above the cut-off for heavy drinking (0.5 ng/mg in samples <3 cm and 1.0 ng/mg in samples 3–6 cm in length) among individuals suspected of alcohol abuse in child protection cases. FAEEs were above the cut-off in 23.7% of self-reported abstainers, 43.6% of self-reported moderate drinkers (<60 g/day) and 77.9% of self-reported excessive drinkers (>60 g/day) [163]. Similar findings in a subsequent study show a relatively low reliability of self-reported drinking patterns. FAEEs in hair show 96% specificity and 77% sensitivity for a cut-off of 1.0 ng/mg [164].

### 5.3. Discussion

While EtG in urine can help identify alcohol consumption for a few days after alcohol clears from the blood, hair EtG provides an exposure indicator for long-term alcohol consumption patterns [44,165]. Using the threshold of 30 pg/mg set off by the Society of Hair Testing, hair EtG has a high PPV but a low NPV in a sample of volunteers with a wide range of alcohol consumption patterns. As such, hair EtG is a good tool for identifying alcohol consumption yet it generally fails at correctly identifying abstainers [146].

The presence of EtG in hair (>7 pg/mg) disproved abstinence in 54.5% of a sample of subjects requested to refrain from alcohol consumption [166]. Subjects’ false statements often lead to a high number of false positives and thus unreliable sensitivity, specificity, PPV and NPV based on self-reported alcohol history [149]. Positive hair EtG (>7 pg/mg) was detected significantly more frequently among patients with ALD in a large sample of 104 patients scheduled to undergo liver transplantation (32% among ALD compared to 7% among non-ALD patients; *p* = 0.002) [151].

In contrast, negative results by FAEEs disproved alcohol abuse in 42.3% (<0.2 ng/mg) of subjects suspected of this behavior in a small sample, while showing moderate drinking in 29.5% (0.2–0.5 ng/mg) and proving chronic excessive drinking in 28.2% (≥0.5 ng/mg) [152]. Double negative or double positive results (EtG >7 pg/mg and FAEEs > 0.2 ng/mg) were found in 72.6% of cases in a small sample of subjects. No linear correlation was found to exist between these two markers [167]. Based on these findings, it is recommended that FAEEs results should only be used to reinforce EtG results due to a high incidence of ambiguous results in classifying individuals according to their alcohol consumption pattern [155,167].

Hair EtG was the only biomarker that can differentiate heavy alcohol consumption from social drinking in a sample of patients whose drinking habits were clinically classified based on their alcohol consumption levels [158]. EtG in hair was the best biomarker for assessing chronic alcohol abuse. Combining EtG with any other biomarker did not improve the diagnostic potential of EtG alone for heavy drinking [147,156].

Hair EtG and FAEEs were in agreement in 75.3% of hair samples belonging to subjects undergoing driving ability examination, workplace testing or in child custody cases, including instances when both biomarkers show abstinence or alcohol consumption [168]. A low to moderate correlation was found between combinations of CDT, γ-GTP, AST, ALT, MCV, EtG and FAEEs in a sample of non-drinkers or social drinkers (<60 g/day) and alcoholics (>60 g/day). Hair EtG shows significant differences between non-drinkers or social drinkers and alcoholics, such that hair EtG can discriminate based on alcohol consumption, using a cut-off value of 60 mg/day [157].

EtG measurements revealed a low level of alcohol consumption during pregnancy in a small sample. Indeed, self-reported alcohol consumption rates were higher than alcohol consumption rates shown by hair EtG levels [169]. The disagreement between self-reports and hair EtG testing can be explained as most women self-reported light drinking (method unable to detect EtG < 5 pg/mg), as well as the fact that hair samples were collected at the end of the pregnancy, while alcohol consumption could have occurred many months before and washed out since [169].

### 5.4. Factors Affecting Hair Ethyl Glucuronide and Fatty Acid Ethyl Esters

Accidental exposure to ethanol can produce results over 1.0 mg/kg in alcohol abstainers [170]. The use of hair biomarkers is generally not suitable to determine absolute abstinence as EtG and FAEEs levels are susceptible to environmental factors and by non-beverage alcohol [171]. This is exemplified in a case report in which a female subject showed sporadically low levels of both FAEEs and EtG. These findings could be interpreted as either low alcohol consumption or abstinence in the presence of environmental factors and use of hair products [171].

Herbal hair tonics may contain EtG. As such, external sources of EtG should be considered, especially if subjects deny alcohol consumption [170,172]. Hair coloring was found to have no effect on EtG levels *in vitro*, while both bleaching and perming decrease EtG levels, largely as a result of chemical degradation [164,173]. Bleaching and dyeing decrease hair EtG levels by up to 20%–40%, likely due to EtG oxidation by H_2_O_2_ [164]. Similarly reduced hair EtG levels in alcohol-dependent patients who bleached or colored their hair compared to those with uncolored/unbleached hair were also found in another study [174]. Thermal hair straightening also reduced EtG levels *in vitro* compared to untreated strands [175]. A single application of cleansing shampoos was not associated with hair EtG loss [176]. Hair spray had no influence on EtG levels, suggesting that external alcohol does not increase hair EtG levels [164]. On the other hand, the use of hair spray is associated with elevated hair FAEEs levels, likely resulting from alcohol in the product. Bleaching and dyeing have no significant effects on hair FAEEs. Due to the contrasting effects of different hair products on EtG and FAEEs, using FAEEs and EtG assessment concomitantly can help protect against false positive FAEEs or false negative EtG [164]. Use of ethanol-containing hair lotions may give rise to false positive FAEEs results [162].

Using dose-adjusted detection times, decreased renal function led to higher levels of hair EtG compared to healthy subjects, although the correlation between hair EtG and the degree of renal dysfunction was weak (*p* = 0.08) [177].

The sample length generally has little effect. Assuming constant alcohol consumption over time, the percentage of hair samples with EtG content >7 pg/mg was constant regardless of sample length in a large library, suggesting there is no substantial washout of EtG from the hair strand over time. However, samples <3 cm in length show unusually high EtG levels, suggesting EtG incorporation from sweat following recent alcohol consumption [178,179]. While using a longer length of hair may offer a long-term assessment of alcohol consumption, different drinking patterns over time may confound results [179].

The method used for sample size reduction influences the amount of EtG that can be extracted from hair samples. For example, milling produced markedly higher percentages of extractable EtG than cutting (137%–230%), regardless of the extent of sample pulverization [180]. A different study showed that extensive sample pulverization increased the amount of extractable EtG compared to cutting or weak pulverization. This study argues that while the Society of Hair Testing provides cut-offs for different drinking patterns, inter-laboratory variability may arise owing to different sample preparation methods [181]. In addition, the method used for washing the sample further determined the amount of extractable EtG and FAEEs [182].

Age, gender and body mass index did not significantly affect the ability of hair EtG to predict alcohol drinking patterns [142,147,183]. However, a relatively high degree of discordance between EtG and FAEEs was found in a sample of females selected for alcohol abuse monitoring. This suggests that these two biomarkers should be used together, particularly among females, where use of hair products may alter hair EtG and FAEEs levels. Using a combination of hair EtG and FAEEs led to the lowest rate of false-negative and false-positive [157,184,185]. Interestingly, seasonal differences were found, corresponding to hair growth patterns during winter, spring, summer and autumn. As such, the highest EtG levels in hair were found during the winter and the lowest during the summer [183]. The subject’s weight did not play a significant effect in FAEEs incorporation in hair. Furthermore, FAEEs incorporation in head hair and non-head hair was similar. However, FAEEs can leach out during hair washing [163].

### 5.5. Ethyl Glucuronide in Other Hair Matrices

A strong correlation was found between head hair EtG levels and self-reported alcohol consumption in a study (r = 0.8921, *p* < 0.0001). Among subjects with negative head hair EtG, negative results were also found in beard, chest, axillary, stomach, arm and leg hair. In contrast, pubic hair was positive for EtG in 45.4% of subjects in which head hair was negative. Positive EtG were found in all matrices in subjects in which head hair was positive. Axillary hair generally has lower EtG levels than head hair, while pubic hair has higher levels [186].

Based on pair comparisons, EtG levels in hair were not significantly different between scalp and either of chest, arm or leg. Good correlations were found between scalp hair and chest, arm or leg hair for samples classified as negative (75%–100% association in scalp hair EtG < 7 pg/mg) and samples classified as chronic excessive drinking (73%–100% association in scalp hair EtG > 30 pg/mg). The correlation was poor for social drinkers (EtG 7–30 pg/mg). Chest, arm and leg hair show >78% sensitivity and >75% specificity for drinking behavior as assessed by scalp hair EtG. EtG levels were low in axillary hair, likely a result of degradation by deodorants or leaching through sweat. EtG levels were high in pubic hair, likely due to incorporation from urine. Chest hair appears to be the best alternative to head hair, although sample size differences exist. When comparing EtG levels in different hair samples belonging to the same individual, one must take into account the time frame represented by the sample length, according to the telogen phases of each sample area [145,154]. In another study, EtG became detectable in daily shaved beard after as little as 9 h following alcohol consumption, and fell below the limit of detection after 8–10 days. Peak levels were reached between days 2–4. This study shows the usefulness of an assay that utilizes a small sample of hair [187].

### 5.6. Ethyl Glucuronide and Ethyl Sulfate in Other Matrices

EtG and EtS were analyzed in blood in several studies. EtG C_max_ in blood was 0.36 mg/L (range 0.28–0.41 mg/L) in healthy volunteers receiving 0.5 g/kg alcohol and 1.06 mg/L (range 0.80–1.22 mg/L) in healthy volunteers receiving 1.0 g/kg alcohol. EtG levels peaked after 3.5 h with 0.5 g/kg and after 5.5 h with 1 g/kg in blood (T_max_) [96]. EtG and EtS were 11.0 mg/L and 3.7 mg/L, respectively, in a blood sample collected >8 h after alcohol consumption in a driver involved in a traffic accident [188]. The EtG concentration in blood ranged from 460–6250 ng/mL (mean 2179 ng/mL, median 1885 ng/mL) and that of EtS from 200–2720 ng/mL (mean 1157 ng/mL, median 1020 ng/mL) in traffic offense cases. In dried blood spots, the EtG concentration ranged from 428–6690 ng/mL (mean 2126 ng/mL, median 1885 ng/mL) and that of EtS from 161–2680 ng/mL (mean 1177 ng/mL, median 1085 ng/mL) [189]. A recent study conducted among individuals injured in traffic accidents or at work showed that EtG or EtS in blood could be detected in up to 17% of the sample, including individuals with negative BAC [190].

A good correlation was observed between EtG levels in nails and self-reported alcohol consumption. The sample size was too small to allow for the calculation of specificity and sensitivity [191]. EtG in nails showed excellent specificity for detecting any alcohol consumption in another study [192]. EtG in saliva was detectable in only one subject at a dose of 0.5 g/kg alcohol. In subjects receiving 1.0 g/kg alcohol, EtG C_max_ in saliva was 0.032 mg/L (range 0.013–0.059 mg/L). EtG levels peaked after 3.5 h [96].

In post-mortem toxicology, the presence of EtG or EtS in urine or blood, coupled with positive alcohol levels, supports ante-mortem alcohol consumption. Alternatively, the absence of EtG or EtS in blood excludes ante-mortem alcohol consumption in alcohol-positive individuals. Alcohol was likely synthesized post-mortem in such individuals, including diabetics. The presence of non-oxidative ethanol metabolites such as EtG and EtS points towards ingestion, thus distinguishing between the two [193,194,195].

EtG concentrations were significantly higher in individuals with a history of alcohol abuse during post-mortem analysis in vitreous humor (4.2 ± 4.8 mg/L, *p* < 0.001), in serum (6.9 ± 8.9 mg/L, *p* < 0.01) and in cerebrospinal fluid (1.7 ± 2.7 mg/L, *p* < 0.01) compared to individuals without a documented history of alcohol abuse [105]. EtG levels in teeth, assessed by LC-MS/MS, can also estimates alcohol use [196].

## 6. Carbohydrate-Deficient Transferrin

Transferrin is a liver glycoprotein made up of a polypeptide chain, two metal ion-binding sites and two N-linked glycan chains. CDT refers to transferrin isoforms lacking one or two complete or incomplete glycan chains, the most common of which are asialotransferrin, monoasialotransferrin and diasialotransferrin. CDT quantification generally refers to diasialotransferrin measurements [197]. Chronic alcohol consumption interferes with the glycosylation of several glycoproteins, including transferrin. Moderate to heavy drinking (50–80 g alcohol/day) for several days decreases the carbohydrate content of transferrin, thus giving rise to free sialic acid and sialic-acid deficient transferrin. CDT is thus a biomarker of moderate to heavy alcohol consumption. CDT levels return to normal within approximately 2 weeks of drinking cessation [82,104,198]. As such, CDT is a useful indirect marker for both initial screening as well as relapse [82]. The diagnostic usefulness of CDT is the same when using absolute or relative values [199]. CDT is generally expressed as the percentage of CDT divided by the amount of total transferrin. Furthermore, the disialotransferrin glycoform provides the most accurate representation of alcohol intake. Glycation of serum transferrin *in vivo* has no influence on CDT levels [102,200,201].

Early studies assessed CDT levels in hospital populations with either suspected alcohol abuse or with conditions not related to alcohol consumption. Each patient's self-reported alcohol consumption was characterized as <60 g/day or >60 g/day, while alcohol intoxication at the time of admission was assessed by breath test. CDT had 70% sensitivity and 98% specificity of identifying alcohol consumption of >60 g/day with a cut-off of 2.4%, regardless of the presence or etiology of liver diseases. The higher incidence of positive CDT results among patients with alcoholic liver diseases than with liver diseases of other etiology suggests continued high alcohol consumption in the former [202]. Furthermore, there were no significant differences between subjects or between groups over time in small samples of healthy male social drinker volunteers receiving 20, 40, 60 or 80 g alcohol/day over a 21 day period, suggesting that CDT is not a good marker for short-term alcohol consumption, even at 80 g/day [203]. Results of recent studies correlating CDT with alcohol consumption patterns are shown in Table 4 [77,90,93,102,105,147,157,158,166,191,204,205,206,207,208,209,210,211,212,213,214].

### 6.1. Discussion

CDT generally correlates well with an individual’s drinking pattern, especially during the preceding 30 days. In a sample of drinkers involved in traffic accidents, CDT in plasma was correlated with the total number of drinks consumed in the past month (r = 0.38, *p* = 0.003) and the total number of heavy-drinking days in the past year (r = 0.48, *p* < 0.001) [122]. Similar associations were also shown elsewhere [209,215].

Serum CDT can differentiate between heavy drinkers and non-drinkers, and between heavy drinkers and social drinkers (*p* < 0.0005 for both), but not between social drinkers and non-drinkers (*p* = 0.063) [158]. Little variation in CDT levels was seen for alcohol consumption below a threshold of 2 drinks/day (6–10 drinks/week), past which point a significant increase was observed (≥11–20 drinks/week) [215]. CDT in serum was the best biomarker for detecting an average consumption of >40 g/day compared to <40 g/day in a large Russian population with high levels of alcohol consumption (67% sensitivity and 71% specificity). CDT did not detect hazardous drinking patterns (<60% sensitivity) [216]. CDT results correlate with AUDIT questionnaires results [217], but lack sufficient sensitivity to detect binge drinking [140,218]. CDT and BAC were significantly correlated in drivers involved in car accidents with BAC >0.5 g/L, suggesting chronic alcohol abuse in this population [219].

Median serum CDT levels measured by HPLC were 0.84% among abstinent or light drinkers (<210 g/week for men and <140 g/week for women) in a study. CDT levels were significantly higher in drivers applying for license regranting after a rehabilitation programme (median 0.90%, IQR 0.80–1.10, 3% of sample positive for CDT), as well as in drivers involved in car accidents with BAC > 0.5 g/L (median 1.20%, IQR 0.90–2.00; 27% of sample positive for CDT) compared to controls (*p* < 0.001) [219]. The incidence of CDT-positive subjects (>1.7%) was 7.5% in a sample of 562 individuals applying for driving license regranting with self-reported alcohol abstinence [220]. CDT levels were not different between first time and recidivist male DUI subjects, using a cut-off of 2.7%. This suggests that CDT cannot be used to predict recidivism among DUI subjects [221].

**Table 4:** Carbohydrate-deficient Transferrin.

Study	Matrix and Method	Study Population and CDT Levels	CDT Associations—Diagnostic Performance
204	Matrix not specified	Healthy Korean subjects	77.8% sensitivity, 70.4% specificity, 19.4% PPV and 97.2% NPV of predicting CDT ≥ 2.47% at a cut-off of 3.38 drinks/week in flushers, 62.2% sensitivity, 69.6% specificity, 24.7% PPV; 92.0% NPV of predicting CDT ≥ 2.47% at a cut-off of 11.25 drinks/week in non-flushers
	Nephelometry	CDT 2.47% achieved after fewer drinks (3.38 drinks/week) in flushers compared to non-flushers (11.25 drinks/week)	
147	Serum	Healthy volunteers	CDT a better predictor of heavy drinking (>60 g/day) than at-risk drinking (>20 g/day for women and >30 g/day for men)
	Capillary zone electropho-resis (CZE)	Mean CDT levels 0.7% ± 0.2% (range 0.2%–1.2%) in teetotalers, 0.8% ± 0.2% (0.5%–1.6%) in low-risk drinkers, and 2.1% ± 2.5% (0.5%–11.8%) in at-risk drinkers	
77	Serum	Subjects examined for driver’s license re-granting	n/a
	CZE	Higher in heavy drinkers than non-drinkers (median 6.7%, IQR 3.2–12.3 *vs.* 1.0%, IQR 0.8–1.9, *p* < 0.0001)	
205	Serum	Subjects undergoing examination for driver’s license regranting	74.6% sensitivity and 99.3% specificity at a cut-off of 1.7%; 48.8% sensitivity and 100% specificity at a cut-off of >2.3% used to characterize drinking relapse
	CZE		
166	Matrix not specified	Subjects required to abstain, Abstinence was disproved in 46.4% by immunoturbidimetry and in 17.8% by HPLC in subjects in which abstinence was previously disproved by EtG in hair (>7 pg/mg)	n/a
	Immuno-turbidimetry (cut-off 2.6%)		
	HPLC (cut-off 1.77%)		
206	Serum	Pregnant women: Detected in 99.3% of sample, 5.3% consistent with possible chronic hazardous drinking (CDT 1.7%–1.9%); 1.3% consistent with probable chronic drinking (CDT ≥ 2%)	n/a
	Capillary electro-phoresis		
90	Serum	Liver transplant patients: 19.8% positive for at least one alcohol biomarker at any visit, with urinary EtG and serum CDT in 93% of cases	25.0% sensitivity, 98.6% specificity, 63.6% PPV and 92.9% NPV of detecting alcohol consumption at a cut-off of 2.6%
	HPLC		
207	Serum	Volunteer liver disease patients classified according to their alcohol intake over the preceding 15 days as either sober (60 g/day) CDT used to discriminate abusers from abstainers	95% specificity and 86% sensitivity, with 96% of subjects correctly classified among non-cirrhotic patients with a cut-off of 1.6%, 83% specificity and 40% sensitivity, with 79% of subjects correctly classified among cirrhotic patients with a cut-off of 1.6%
	CZE		
208	Serum	Homeless individuals: CDT associated with alcohol abused assessed by self-reported drinking patterns according to the FAST Alcohol Screening Test	45% sensitivity and 93% specificity for identifying risky drinking behavior using a cut-off of 2.6%
	Method not specified		
158	Serum	1.9% ± 1.6% in the overall population, 1.3% ± 0.3% in non-drinkers, 1.6% ± 0.8% in social drinkers and 2.7% ± 2.4% in heavy drinkers	50.8% sensitivity and 90.5% specificity for chronic heavy drinking with a cut-off of 2.0%
	HPLC		
209	Plasma	HIV-positive population receiving antiretrovirals with self-reported alcohol consumption patterns, Alcohol consumption detected by CDT in 6.7% of patients reporting alcohol abstinence and in 16.3% of patients reporting any alcohol consumption in the past 30 days	CDT positivity (≥1.8%) significantly associated with the number of drinking days (*p* < 0.05) and the total number of drinks (*p* < 0.05), and marginally with the number of drinks/day (*p* = 0.09)
	HPLC		
210	Serum	HIV-positive heavy drinkers with self-reported alcohol consumption patterns	28% sensitivity, 90% specificity, 60% PPV and 70% NPV for at-risk drinking (≥4 drinks/day or ≥7 drinks/week for women and ≥5 drinks/day or ≥14 drinks/week for men during the past 30 days)—cut-off of >2.6% 36% sensitivity, 88% specificity, 35% PPV and 88% NPV for heavy drinking (≥4 drinks/day for women ≥5 drinks/day for men for at least seven days during the past 30 days) with a cut-off of >2.6%; 41% sensitivity and 86% specificity for frequent heavy drinking (heavy drinking for ≥7 consecutive days) with a cut-off of >2.6%
	Turbidimetric immunoassay		
157	Serum	Social and heavy drinkers undergoing alcohol or drug treatment 2.6% ± 80% in the overall sample; 1.8% ± 23% in non-drinkers or social drinkers (60 g/day)	79.6% sensitivity and 91.0% specificity to discriminate based on alcohol consumption at a cut-off of 2.15%
	Method not specified		
211	Serum	Higher in subjects with likely hazardous alcohol consumption by AUDIT(average 44.2 ± 12.2 drinks/week) than matched teetotalers (5.1% ± 3.6% *vs.* 1.9% ± 0.9%, *p* < 0.001)	84% sensitivity, 92% specificity, 91.3% PPV and 85.2% NPV diagnosing likely hazardous alcohol consumption at an optimal cut-off of 2.4%
	Turbidimetric immunoassay		
191	Serum	Suspected chronic excessive alcohol consumption CDT levels negative in 100% of subjects deemed eligible for driving/firearm license renewal, adoption or liver transplant by a physician (median 1.25%, min 0.5% and max 2.6%) CDT positive in 26.9% of subjects deemed not eligible (median 1.85%, min 0.7% and max 26.5%)	27% sensitivity and 100% specificity for assessing chronic alcohol abuse with a cut-off of 2.7%
	Multi-capillary electro-phoresis		
93	Serum	Subjects undergoing alcohol withdrawal, Wide range on the day of admission (1.2%–73.0%)	99% specificity and 35% sensitivity to identify social drinking with a cut-off of 1.6%
	CZE		
102	Serum	Outpatients treated for alcohol-related problems, 0.87%–6.9% (mean 2.1%, median 1.4%) at baseline, 35% above CDT 1.7% reference cut-off for alcohol abuse, 30% above CDT 1.9%	n/a
	HPLC		
212	Serum	Alcohol-dependent inpatients: 86.2% had CDT levels >2.6% cut-off of alcohol abuse	n/a
	Immunoassay		
213	Serum	Alcohol-dependent patients: Mean CDT levels 1.82% (range 1.40%–2.54%), Mean CDT absolute value 43.1 mg/L (range 23.0–61.1 mg/L)	87.3% sensitivity, 96.2% specificity, 98.2% PPV and 75.8% NPV to detect alcohol abuse compared to social drinking using an absolute cut-off of 58.4 mg/L 88.9% sensitivity, 94.2% specificity, 97.4% PPV and 7.8% NPV to detect alcohol abuse compared to social drinking using a relative cut-off of 2.29%
	Immunoassay		
105	Cerebrospinal fluid, serum or vitreous-Immunoassay	Post-mortem analysis: Higher in subjects with a history of alcohol abuse *vs.* control (4.3% ± 2.1% *vs.* 2.3% ± 0.6%, *p* < 0.05)	64% sensitive and 100% specificity for alcohol abuse with CDT cut-off of 3.4% in cerebrospinal fluid No association in serum or vitreous humor
214	Blood sample	Post-mortem analysis: Negative in 71.0% of non-drinkers (reported by someone else) and controls (proved): Positive in 53.8% of drinkers	59% sensitivity and 71% specificity for past 15 days alcohol consumption prior to death in subjects with suspected alcohol abuse
	ISEFE in polyacryl-amide gel		

CDT—carbohydrate-deficient transferrin; CZE—Capillary zone electrophoresis; EtG—ethyl glucuronide; IEFE—Isoelectro-focusing electrophoresis; IQR—interquartile range; HPLC—high-performance liquid chromatography; NPV—negative predictive value; PPV—positive predictive value.

During post-mortem analysis, positive CDT was found in 60.0% of samples with positive BAC. Of these, 30% had BAC >1‰ (positive CDT in 100.0%), and 70% had BAC <1‰ (positive CDT in 42.8%). Positive CDT was found in 66.7% of individuals with severe liver disease [214].

CDT can be further used in populations required to remain abstinent, such as liver transplant patients and pregnant women. Serum CDT assayed by double antibody radioimmunoassay had 92% sensitivity and 98% specificity for detecting alcohol relapse in a sample of subjects who underwent orthotopic liver transplant for alcoholic cirrhosis [222]. In a sample of orthotropic liver transplantation candidates with alcoholic liver cirrhosis, only 30.2% admitted drinking in the past 6 months, yet 61.9% were positive for at least one alcohol biomarker (hair EtG, urine EtG, BAC, methanol or CDT). Of patients denying alcohol consumption in the preceding 6 months, 8.3% showed positive blood CDT (≥5 mg/L). As serum CDT is a poor biomarker for low level alcohol consumption, self-reported abstinence in these individuals can be disproved by other, more sensitive methods [149]. CDT had low sensitivity in a sample of patients with a self-reported history of sustained heavy alcohol consumption, as CDT results may be confounded by such factors as cirrhosis and obesity, especially among females [223]. In another sample of liver transplant patients, alcohol consumption was self-reported by only 3.6% of subjects, yet almost 20% were shown to consume some alcohol with the help of biomarkers (urinary EtG, BAC, methanol, CDT, ALT, AST, γ-GTP and MCV) [90].

In a sample of pregnant women, 12.3% continued drinking during pregnancy, with 4.8% reporting infrequent binge drinking. Self-reported drinking during pregnancy was associated with AUDIT scores. However, none of the subjects reporting drinking during pregnancy tested positive for CDT in serum by HPLC (<1.7% disialotransferrin). This reflects relatively infrequent and low alcohol consumption during pregnancy [140].

The fate of CDT in patients beginning alcohol withdrawal treatment was also assessed. A wide range of CDT values were recorded on the day of admission in subjects undergoing alcohol withdrawal. The relative concentration of CDT (%CDT) at study entry were higher in alcohol-dependent males than females (5.67% ± 0.74% *vs.* 3.22% ± 0.37%, *p* = 0.027), although daily alcohol consumption was comparable (197.0 ± 17.14 g/day *vs.* 159.4 ± 21.19 g/day). CDT levels decline rapidly within the first 4 days of treatment, and significant differences can be seen upon completion of detoxification. The percentage of patients with CDT >2.6% generally declined over the treatment period. However, 34.5% of patients continued to have CDT >2.6% for up to 6 weeks into the study, suggesting that CDT data needs to be interpreted with care when abstinence is required [93,212,224]. Disialotransferrin was found to have a half-life of 8.5–15 days (mean 12.6 days, median 13.9 days) [102].

### 6.2. Factors Affecting CDT

CDT levels were significantly associated with the body mass index (*p* = 3.71 × 10^−9^), female gender (*p* = 2.30 × 10^−9^) and smoking (*p* = 8.28 × 10^−8^), but not with age [215,219]. The usefulness of CDT is reduced in overweight or obese subjects, as CDT levels are lower in these compared to lean individuals consuming comparable amounts of alcohol [225,226]. In contrast, CDT levels are higher in smokers compared to non-smokers consuming comparable levels of alcohol [225].

CDT was detected in almost 100% of samples belonging to pregnant women in a study, with low incidences consistent with hazardous alcohol consumption [206]. CDT in serum was analyzed by HPLC in a sample of pregnant women self-reporting alcohol abstinence. Drinking was excluded by negative urine and serum EtG in all subjects. Absolute CDT (disialotransferrin) levels, transferrin levels and %CDT were 5.2 mg/dL (IQR 3.6–6.7 mg/dL, min 1.3 mg/dL, max 10.2 mg/dL), 378 mg/dL (IQR 310–424 mg/dL, min 221 mg/dL, max 681 mg/dL) and 1.4% (IQR 1.1%–1.6%, min 0.5%, max 2.0%), respectively. Transferrin values correlate well with both CDT (disialotransferrin) levels (r = 0.89, *p* < 0.001) and %CDT (r = 0.66, *p* < 0.001). Both transferrin (r = 0.68, *p* < 0.001) and CDT levels (r = 0.77, *p* < 0.001) correlate with gestational week. Likewise, %CDT were different between women in the first trimester (mean 1.01% ± 0.19%), second trimester (mean 1.30% ± 0.14%) and third trimester (mean 1.53% ± 0.22%) (*p* < 0.001). In two subjects, %CDT levels in the third trimester approached the cut-off value of 2.0% for chronic alcohol use even though they were abstinent [227]. Similar findings are reported in a separate study [228]. Disialotransferrin, the CDT species most often analyzed, shows a high degree of increase during pregnancy (1.07% ± 0.17% baseline to 1.61% ± 0.23% before delivery). CDT levels return to normal in the post-partum stage [228]. CDT levels are decreased in postmenopausal women compared with women at the fertile stage [229,230]. The effects of contraceptives are not well established [229,230,231].

Measurements of CDT have been proposed for the diagnosis of alcoholic liver disease. However, CDT elevations can occur in sepsis, anorexia nervosa, and airway diseases [232]. Lower values of sensitivity have also been reported with iron overload [233]. Although CDT is usually unaffected by the presence of liver disease, false positive results have been reported in patients with primary biliary cirrhosis and with severe non-alcohol-related hepatic failure [234,235]. Therefore, individuals with suspected primary biliary cirrhosis who are positive for CDT should be evaluated further by using mitochondrial autoantibodies to pyruvate dehydrogenase [236]. As a result, the utility of CDT testing depends upon the clinical picture and other biochemical tests [237].

CDT performs better in non-cirrhotic than in cirrhotic patients based on self-reported alcohol consumption in the past 15 days [207]. CDT in serum was analyzed by capillary electrophoresis and by nephelometry in a sample of healthy controls (<25 g alcohol/day), abstinent patients with liver disease, alcoholic patients with liver disease, and individuals consuming varying amounts of alcohol. Among abstinent individuals, CDT levels were higher in those with liver diseases compared to controls (0.9% *vs.* 0.5%, *p* = 0.046). Furthermore, CDT levels were higher in individuals consuming >60 g alcohol/day than those consuming <60 g alcohol/day (*p* = 0.034). CDT levels were slightly lower when assessed by capillary electrophoresis, but a good correlation was found between this method and nephelometry [238]. Hepatitis C virus seropositivity is associated with significantly decreased baseline CDT levels in patients undergoing treatment for alcohol dependence [239]. Absolute CDT values are not affected by liver disease, yet the relative values are. Relative CDT values are highest in patients with alcoholic hepatitis, and lowest in primary biliary cirrhosis patients [213]. In a different study, CDT levels assessed by high-performance liquid chromatography (Helander HPLC) were associated with binge drinking behavior in adolescents with alcohol abuse, while no differences were found between binge drinkers and non-binge drinkers when CDT levels were assessed by immunonephelometric assay (N Latex) [240].

Genetics also play an important role. A CDT indicative of alcohol abuse (2.47%) was achieved after fewer drinks in a sample of Korean subjects who experience facial flushing after drinking, associated mainly with acetaldehyde accumulation, compared to non-flushers [204]. CDT measurements are also influenced by CDT hereditary syndrome [241,242,243,244,245]. Transferrin CD variants may further complicate results, particularly when quantified by liquid chromatography methods [246]. CDT measurements in serum were affected by a T139M transferrin variant. The presence of this variant in a subjects suspected to suffer from alcoholism led to unquantifiable CDT levels by HPLC and capillary zone electrophoresis and low levels for isoelectric focusing. CDT was accurately quantified by immunoassay [247].

## 7. Conclusions

Based on the various laboratory tests employed, the array of findings can delineate the specific amount and the period when alcohol was consumed in individuals with alcohol problems. However, many factors can influence the analytical performance of the tests. This review provides clinicians with tests that will accurately detect heavy drinking despite denial by the patient. The role of the laboratory is to promote assay standardization and aid in results interpretation with the intent of guiding the medical professional toward the proper use of a specific laboratory test in a specific time frame in order to meet the clinical need. Clinical management of pharmacotherapy with drugs of use in patients denying drinking is challenging due to inter-individual variability in alcohol metabolism. Therefore, initiating any therapy in this population requires an interdisciplinary team that includes clinicians, the laboratory, and the patient.

## Abbreviations

ADH: alcohol dehydrogenase
ALDH: aldehyde dehydrogenase
ALT: alanine aminotransferase
AST: aspartate aminotransferase
AUDIT: Alcohol Use Disorders Identification Test
BAC: blood alcohol concentration
BrAC: breath alcohol concentration
CDT: carbohydrate-deficient transferrin
C_max_: maximum concentration
CYP2E1: cytochrome p450 2E1
DUI: driving under the influence
EtG: ethyl glucuronide
EtS: ethyl sulfate
FAEE: fatty acid ethyl ester
γ-GTP: γ-glutamyl transpeptidase
HPLC: high-performance liquid chromatography
IQR: interquartile range
LC-MS: liquid chromatography-mass spectrometry
LC-MS/MS: liquid chromatography-tandem mass spectrometry
MCV: mean corpuscular volume
NPV: negative predictive value
OR: odds ratio
PEth: phosphatidylethanol
PPV: positive predictive value
T_max_: time to achieve maximum concentration
